# Neuronatin deletion causes postnatal growth restriction and adult obesity in 129S2/Sv mice

**DOI:** 10.1016/j.molmet.2018.09.001

**Published:** 2018-09-15

**Authors:** Steven J. Millership, Simon J. Tunster, Mathew Van de Pette, Agharul I. Choudhury, Elaine E. Irvine, Mark Christian, Amanda G. Fisher, Rosalind M. John, James Scott, Dominic J. Withers

**Affiliations:** 1 MRC London Institute of Medical Sciences, Du Cane Road, London, W12 0NN, UK; 2 Institute of Clinical Sciences, Faculty of Medicine, Imperial College London, Du Cane Road, London, W12 0NN, UK; 3 School of Biosciences, Cardiff University, Museum Avenue, Cardiff, CF10 3AX, UK; 4 Institute of Reproductive and Developmental Biology, Department of Surgery and Cancer, Imperial College London, Du Cane Road, London, W12 0NN, UK; 5 National Heart and Lung Institute, Department of Medicine, Imperial College London, Du Cane Road, London, W12 0NN, UK

**Keywords:** Obesity, Postnatal growth, Imprinted genes, Neuronatin, Genetic background, Energy homeostasis, WAT, white adipose tissue, BAT, brown adipose tissue, SPC, signal peptidase complex, PEG, paternally expressed gene, HFD, high fat diet, GH, growth hormone, CLAMS, comprehensive lab animal monitoring system

## Abstract

**Objective:**

Imprinted genes are crucial for the growth and development of fetal and juvenile mammals. Altered imprinted gene dosage causes a variety of human disorders, with growth and development during these crucial early stages strongly linked with future metabolic health in adulthood. Neuronatin (*Nnat)* is a paternally expressed imprinted gene found in neuroendocrine systems and white adipose tissue and is regulated by the diet and leptin. Neuronatin expression is downregulated in obese children and has been associated with stochastic obesity in C57BL/6 mice. However, our recent studies of *Nnat* null mice on this genetic background failed to display any body weight or feeding phenotypes but revealed a defect in glucose-stimulated insulin secretion due to the ability of neuronatin to potentiate signal peptidase cleavage of preproinsulin. *Nnat* deficiency in beta cells therefore caused a lack of appropriate storage and secretion of mature insulin.

**Methods:**

To further explore the potential role of *Nnat* in the regulation of body weight and adiposity, we studied classical imprinting-related phenotypes such as placental, fetal, and postnatal growth trajectory patterns that may impact upon subsequent adult metabolic phenotypes.

**Results:**

Here we find that, in contrast to the lack of any body weight or feeding phenotypes on the C57BL/6J background, deletion of *Nnat* in mice on 129S2/Sv background causes a postnatal growth restriction with reduced adipose tissue accumulation, followed by catch up growth after weaning. This was in the absence of any effect on fetal growth or placental development. In adult 129S2/Sv mice, *Nnat* deletion was associated with hyperphagia, reduced energy expenditure, and partial leptin resistance. Lack of neuronatin also potentiated obesity caused by either aging or high fat diet feeding.

**Conclusions:**

The imprinted gene *Nnat* plays a key role in postnatal growth, adult energy homeostasis, and the pathogenesis of obesity via catch up growth effects, but this role is dependent upon genetic background.

## Highlights

• Deletion of the imprinted gene neuronatin in mice alters postnatal growth.
• This postnatal growth defect and subsequent catch up growth is background-specific.
• Leads to reduced energy expenditure, hyperphagia and leptin resistance in adulthood.
• *Nnat* null mice are more susceptible to obesity via aging or high fat diet feeding.
• Defects in normal insulin and growth hormone secretion may underpin these findings.

## 1 Introduction

Genomic imprinting results in monoallelic expression of a subset of mammalian genes specified by their parent-of-origin [1]. Many of these ∼150 genes are critical not only for placental function and normal fetal growth and development [2], [3], [4], [5] but also in a wide range of processes vital for the survival and development of neonates including thermoregulation, feeding behavior, and regulation of glucose and lipid metabolism [6], [7], [8], [9], [10], [11], [12], [13], [14]. The most widely accepted theory for the existence of genomic imprinting is the ‘parental conflict’ hypothesis, which predicts that females, who are equally related to all her offspring, maximize reproductive longevity by conserving resources over multiple litters and pups. In contrast, with possible multiple paternity across litters, males are related to just some of the offspring of a specific mother; therefore, it is in his genetic interest for his offspring to extract maximal maternal resources [15]. The parental conflict hypothesis is consistent with the observed silencing of growth restriction genes by the paternal genome and silencing of growth promoting genes by the maternal genome [16]. Highlighting the importance of imprinted gene dosage, human genetic disorders and mouse models with altered imprinted gene expression are associated with a wide range of diseases ranging from psychiatric conditions and cancer to metabolic disorders such as obesity and diabetes [17], [18], [19], [20], [21], [22], [23], [24], [25]. Additionally, control of growth and development by imprinted genes in the postnatal period strongly influences adult health status [26], [27].

Neuronatin (*Nnat*) (also known as *Peg5*) is a paternally expressed imprinted gene found in neuroendocrine systems including the hypothalamus, pancreatic beta cells, and pituitary as well as in adipose tissue [28], [29], [30], [31], [32]. *Nnat* expression peaks in the developing fetus and the immediate postnatal period but is also maintained throughout adulthood in mice at these sites [29], [33], [34], [35]. Unlike the majority of imprinted genes, which are found in imprinting clusters, the *Nnat* gene resides in a ‘microimprinted’ region within the intronic sequence of the neighboring biallelic gene *Blcap*, with differential *Nnat* expression likely controlled by localized methylation of the silenced maternal allele [32], [36], [37], [38]. *Nnat* expression is acutely regulated by nutrient status in metabolic tissues, and by leptin in various hypothalamic nuclei that are key to controlling feeding behavior and energy expenditure [28], [39], [40], [41]. *Nnat* expression has also been associated with a TRIM28-dependent mechanism thought to contribute to the stochastic obesity seen in mice on some genetic backgrounds [42]. Single nucleotide polymorphisms in the human *NNAT* locus are associated with extreme childhood obesity and reduced expression of *NNAT* is found in human adipose tissue in obese children. Together, these data suggested a role for neuronatin in the regulation of body weight and adiposity potentially via effects upon feeding behavior [28], [42], [43].

We have recently reported that in pancreatic beta cells NNAT interacts with the signal peptidase complex (SPC) on the endoplasmic reticulum membrane, facilitating translocation of nascent preproinsulin prior to its cleavage by the SPC. Mice with paternal-deletion of *Nnat* on both the 129S2/Sv and C57BL/6J genetic backgrounds display reduced beta cell storage and secretion of mature insulin due to defects in early peptide handling. Blunting of *in vivo* glucose-stimulated insulin secretion resulted in perturbed glucose homeostasis under conditions of nutrient-excess in these null mice on a C57BL/6J background [41]. However, on this genetic background we did not detect significant changes in body weight and related phenotypes such as feeding in mice with global deletion of *Nnat* either at the postnatal stage or in adulthood. In view of this somewhat surprising result, we have now undertaken extensive longitudinal analysis of the effects of *Nnat* deficiency in 129S2/Sv mice. This genetic background, possibly due to strain differences in early development, has historically been shown to reveal roles for both imprinted and non-imprinted genes in terms of early growth, subtle phenotypes that may be obscured on the obesity-prone C57BL/6J background [44], [45], [46], [47], [48]. In our current studies, we found that deletion of *Nnat* led to a transient postnatal growth restriction followed by catch up growth after weaning, despite normal weight *in utero* on a 129S2/Sv background. In adulthood, *Nnat* null mice displayed decreased energy expenditure, blunted leptin sensitivity, and hyperphagia, resulting in obesity under high fat diet-feeding or associated with aging. Together, these studies indicate the importance for *Nnat* function, possibly due to its modulation of signal peptide processing, during postnatal growth and the effect that this early-life adversity has on key processes governing feeding and energy homeostasis in adulthood.

## 2 Methods and materials

### 2.1 Animals and diets

ARRIVE guidelines were used for the designing and reporting of animal experiments. When possible, investigators were blinded to genotype of both live animals and tissue/blood samples. For experiments involving feeding studies, mice were randomized by genotype for study groups, and a crossover design was used. All metabolic studies were replicated in at least two independent cohorts.

Generation and maintenance of mice with global deletion of *Nnat* have been described previously [41]. This line was backcrossed in our transgenic animal facility >8 generations onto the 129S2/Sv (Envigo) background before intercrossing of mutant animals for generation of experimental cohorts. Experimental cohorts of group housed, mutant male (unless otherwise stated) mice, and their control littermates at ages described in the text were maintained on a 12-hour light/dark cycle with free access to water and standard mouse chow (RM3, Special Diet Services) and housed in specific pathogen-free barrier facilities. All animal work was carried out in accordance with the UK Animals (Scientific Procedures) Act (1986) as well as being approved by the Imperial College Animal Welfare and Ethical Review Body and by the UK Home Office. For high fat diet (D12451, 45% energy from fat, Research Diets) feeding, animals were fed diet from 10 weeks of age. Embryonic and placental wet weights were taken at the stated time points after the formation of a discernible plug. Yolk sac DNA was used for genotyping.

### 2.2 Metabolic analysis

Measurement of body weights and tail vein blood collection were performed as previously described [49]. Levels of serum leptin were determined by ELISA (Millipore). Fat mass was quantified using an EchoMRI Quantitative Whole Body Composition analyzer (Zinsser Analytic) on unanesthetized animals. Assessment of food intake and leptin sensitivity was performed in singly housed mice for 3 consecutive experimental days as previously described [49] using 1.5 mg/kg recombinant mouse leptin (R&D Systems).

Metabolic rate was measured by indirect calorimetry with the use of a comprehensive lab animal monitoring system (CLAMS, Columbus Instruments). Mice were singly housed, with energy expenditure (using constants according to [50]) determined over a 96-hour period. Core body temperature was assessed using a rectal thermometer (MM2000 digital thermometer, TME Thermometers).

For stimulation of growth hormone secretion (GH) *in vivo*, overnight fasted mice were given a single intraperitoneal injection of insulin (0.4 IU/kg) the following morning to induce hypoglycemia. Tail vein blood was collected prior to, and 1 h after, the insulin injection. GH levels in whole pituitary and in serum were assessed by ELISA (Millipore).

### 2.3 Biochemical determination of placental glycogen concentration

Glycogen was extracted from whole placenta, resuspended in distilled H_2_O, and glycogen concentration quantified as described in [46], [51].

### 2.4 Histological techniques

Placentas and white adipose tissue (WAT) were fixed overnight in phosphate-buffered 4% paraformaldehyde (PFA), dehydrated in ethanol, embedded in paraffin, and 10 μm sections were cut on a microtome (Leica). Sections were stained with hematoxylin and eosin (H&E) [46], and light microscope images were used to measure adipocyte area with ImageJ. Four randomly selected fields of view were used per section, with two sections taken at four different medial points through the embedded tissue. Data were averaged per medial point, and all counts were performed blindly.

### 2.5 Expression analysis

For separation of the preadipocyte-containing stromal cell fraction and mature adipocytes from white adipose tissue, epididymal fat depots were digested with collagenase (1 mg/mL, Sigma–Aldrich) at 37 °C in Krebs-HEPES-Bicarbonate buffer and centrifuged, as previously described [52]. Tissues were homogenized directly into Trizol reagent for RNA analysis or lysis buffer (50 mM Tris-HCl pH 7.5, 150 mM NaCl, 1% Triton X-100, 1 mM EDTA with protease inhibitors (Complete mini from Roche)) for protein work. mRNA was extracted using RNeasy kits (Qiagen), normalized for cDNA synthesis, and expression assessed by quantitative RT-PCR using Taqman reagents on a 7900HT Real Time PCR cycler. A list of probes used (Applied Biosystems) can be found in Supplemental Table 1. Protein expression was analyzed by western blotting from clarified lysates normalized for protein content by BCA method (Bio-Rad), all as previously described [49] using antibodies against NNAT (Abcam, ab27266).

### 2.6 PCR analysis of placental *PEG* expression

Pooled cDNA from placenta of at least four wild type (129S2/Sv) conceptuses at various time points, and also from whole brain at P0 were amplified by standard PCR using primers for *Nnat* and other paternally expressed genes listed in Supplemental Table 2.

### 2.7 Statistical analyses

Data sets were analyzed for normal distribution using the D'Agostino-Pearson normality test. Statistical significance between groups was determined using GraphPad Prism 7 by 2-tailed Student's *t* test, Mann–Whitney U test or ANOVA. The Mann–Whitney *U* test, or Kruskal–Wallis test were used as a nonparametric equivalent. Bonferroni (or Dunn's for nonparametric) and Geisser-Greenhouse post hoc tests to correct for multiple comparisons and repeated measures were performed where required. Significance of the difference in observed vs expected appearance of a particular genotype was determined using the chi-squared test. A probability of error less than 5% was considered significant (i.e., *p* < 0.05) and general statistical information for individual experiments (*p* values and *n* numbers) can be found in the figure legends.

## 3 Results

### 3.1 *Nnat* deficient juveniles on a 129S2/Sv background have reduced growth rates in the nest

Recently, global deletion of *Nnat* was shown to cause hypervariable adiposity in adult mice on C57BL/6J background, with a ‘bimodal’ distribution of body weights and two subpopulations of normal and obese mutant animals [42]. Our adult (12 week old) cohorts of *Nnat*^+/−p^ mice on C57BL/6J background showed a similar bimodal body weight distribution but with a subpopulation of lean, rather than obese, *Nnat* null animals, which was not evident upon maternal deletion (Supplemental Figure 1A-C). Relative leanness in this subpopulation of paternally-deleted mice on C57BL/6J background was at least partly due to a reduction in adiposity with no differences (or evidence of bimodality) in terms of food intake or sensitivity to exogenous leptin (Supplemental Figure 1D, [41]). Despite this bimodal distribution in *Nnat*^+/−p^ mice on C57BL/6J background, this did not significantly influence body weight up until 20 weeks of age, either on standard chow or after 20 weeks on a high fat diet (HFD) (Supplemental Figure 1E, [41]). This lack of striking body weight phenotype led us to assess these parameters in *Nnat*^+/−p^ mice on 129S2/Sv background. However, a bimodal body weight distribution was not present in 12 week old *Nnat*^+/−p^ mice on 129S2/Sv background (Supplemental Figure 1F, G).

**Figure 1:**
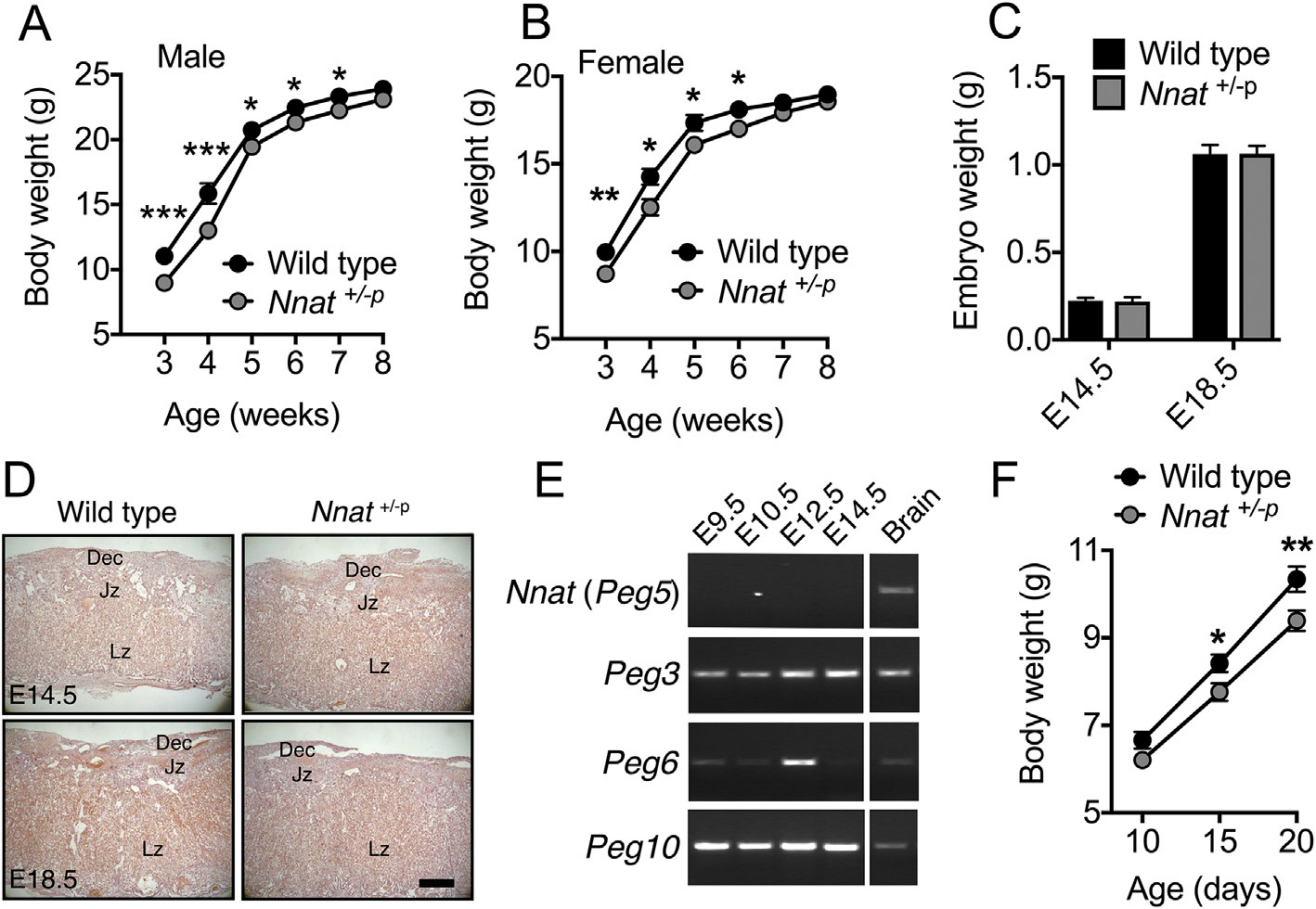
Juvenile *Nnat* deficient mice on a 129S2/Sv background display a ‘catch up’ growth phenotype. (**A, B**) Body weights of male (A) and female (B) wild type and *Nnat*^+/−p^ (paternal deletion) mice from weaning age (3 weeks) (*n* = 19 and 17 for wild type and *Nnat*^+/−p^ mice respectively, *n* = 17 and 18 for females). Data is represented as mean ± SEM. (**C**) Wet weights of wild type and *Nnat*^+/−p^ embryos at E14.5 and E18.5 timepoints (*n* = 22 and 26 for E14.5 and E18.5 respectively per genotype). Data represented as mean ± SD. (**D**) Representative H&E-stained sections through placenta taken from wild type and *Nnat*^+/−p^ embryos at E14.5 and E18.5 (*n* = 5 mice per genotype). Scale bar = 500 μm. Dec = maternal decidua, Jz = junctional zone, Lz = labyrinth. (**E**) PCR analysis of *Nnat* (*Peg5*), *Peg3*, *Peg6* and *Peg10* expression in pooled cDNA extracted from at least four wild type 129S2/Sv placentas between embryonic stages E9.5–14.5. Amplification in pooled cDNA from four 129S2/Sv P0 whole brains served as a positive control. (**F**) Body weights of wild type and *Nnat*^+/−p^ male mice from 10 days old (P10) until weaning age (P20) (*n* = 17 and 20 mice for wild type and *Nnat*^+/−p^ mice respectively). Data represented as mean ± SEM (*p < 0.05, **p < 0.01, ***p < 0.001).

In view of the key roles of imprinted genes in placental, fetal, and early postnatal growth and their influence on metabolic phenotypes in adulthood via such roles, we next undertook a series of studies to understand the role of neuronatin in these processes. At weaning (3 weeks old, P20), both male and female paternally-deleted *Nnat* (*Nnat*^+/−p^) mice on a 129S2/Sv background had significantly lower body weights (Figure 1A,B) but by early adulthood (10 weeks old, P70) this phenotype had disappeared with male and female mutant mice showing no difference in body weight compared to wild type controls (Supplemental Figure 1H, I). This growth restriction in juvenile mice and subsequent post-natal catch up growth on a 129S2/Sv background was absent in mice on a C57BL/6J background [41]. To assess whether reduced growth *in utero* contributed to the observed early postnatal growth restriction, we measured fetal weights and assessed placental structure at embryonic day (E) 14.5 and E18.5. There was no evidence for the loss of *Nnat* deficient mice *in utero* (wild type pups at P14, observed 75, expected 71.5; *Nnat*^+/−p^ observed 68, expected 71.5; χ^2^ = 0.3427, mean litter size = 6.8 ± 0.8, *n* = 21 litters). There was also no evidence of fetal growth restriction at either embryonic stage, and placental weight was normal at both time points, with no overt effect on placental structure apparent from H&E stained midline sections (Figure 1C,D and Supplemental Figure 1J). Placental glycogen, which is proposed to provide a source of energy for fetal growth during late gestation [53], was also unaltered at both time points (Supplemental Figure 1K). The absence of a placental phenotype in *Nnat*^+/−p^ conceptuses is consistent with the observation that unlike the paternally expressed genes *Peg3*, *Peg6*, and *Peg10*, *Nnat* expression was not detectable in the mouse placenta between E9.5 and E14.5 (Figure 1E). In the absence of any signs of fetal growth restriction, we next recorded body weights of *Nnat* deficient pups between P10, when pups were genotyped, and weaning age (P20). We observed a clear reduction in body mass that was statistically significant at P15, and more pronounced at P20 (Figure 1F), suggesting a progressive failure to gain weight. Overall it appears that deletion of *Nnat* on a 129S2/Sv background causes an early postnatal growth restriction in mice that is independent of fetal growth abnormalities.

### 3.2 Restricted growth in *Nnat* deficient juveniles is associated with reduced adipose accumulation

**Figure 2:**
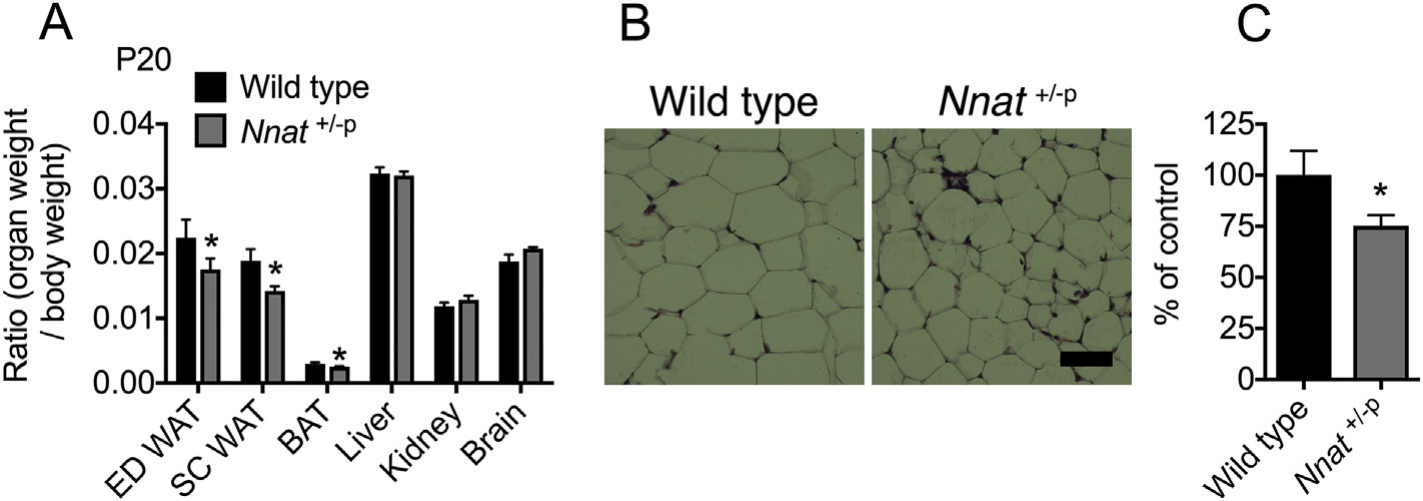
**Growth restricted *Nnat* deficient juveniles accumulate less adipose tissue.** (**A)** Organ to body weight ratios of wild type and *Nnat*^+/−p^ mice at weaning age (P20) ED = epididymal, SC = subcutaneous (*n* = 5 mice per genotype). (**B**) Representative H&E-stained sections through subcutaneous WAT taken from wild type and *Nnat*^+/−p^ mice at weaning age (P20). Scale bar = 50 μm. (**C**) Quantification of images shown in **B** represented as percentage adipocyte size compared with control (wild type) mice (*n* = 5 mice per genotype). In all panels, data represented as mean ± SEM (*p < 0.05).

In the first few days of postnatal life, mice receive mainly fat-based nutrition through milk feeding, to support early growth and metabolism. Between P10 and P21, during which point mice begin to leave the nest, juveniles start to accumulate adipose depots, and therefore require a switch to a lipogenic state in which carbohydrate becomes the major source of fuel [11], [54]. *Nnat* is highly expressed in adipose tissue and has been previously reported to potentiate adipogenesis [29]. At P20, even when normalized to body weight, *Nnat*^+/−p^ mice had reduced fat pad mass in two distinct white adipose tissue (WAT) depots (epididymal and femoral subcutaneous) and reduced interscapular brown adipose tissue (BAT) mass, indicating disproportionate reductions in adipose tissue (Figure 2A). In contrast, although other major organs including the liver, kidney, and brain were significantly lighter in *Nnat*^+/−p^ mice, they remained in proportion to overall body weight (Figure 2A). Reduced WAT mass appeared to be due to smaller adipocytes, suggesting a defect in early lipid storage and WAT accumulation (Figure 2B,C). Previous work has reported that RNAi knockdown of *Nnat* expression in primary mouse adipocytes promotes a thermogenic ‘browning’ response, with increased expression of *Ucp1* and oxidative phosphorylation genes *Pgc1a*, *Cox8b*, and *Cox4* [55]. We found that neuronatin is expressed in mature white adipocytes but not BAT, and that core body temperature in P20 *Nnat*^+/−p^ mice was unaltered, suggesting that reduced body fat in these animals was not caused by an increase in thermogenesis (Supplemental Figure 2A, B). In agreement with this, we found no differences in expression of adipocyte thermogenic markers *Ucp1*, *Pgc1a*, *Prdm16*, and *Dio2* in either BAT or subcutaneous WAT of *Nnat*^+/−p^ mice (Supplemental Figure 2C, D). Overall, relative leanness in *Nnat* deficient juveniles appears to result from reduced adipose tissue accumulation during this early stage.

### 3.3 Adult *Nnat* deficient mice have reduced energy expenditure and display leptin resistance and hyperphagia

**Figure 3:**
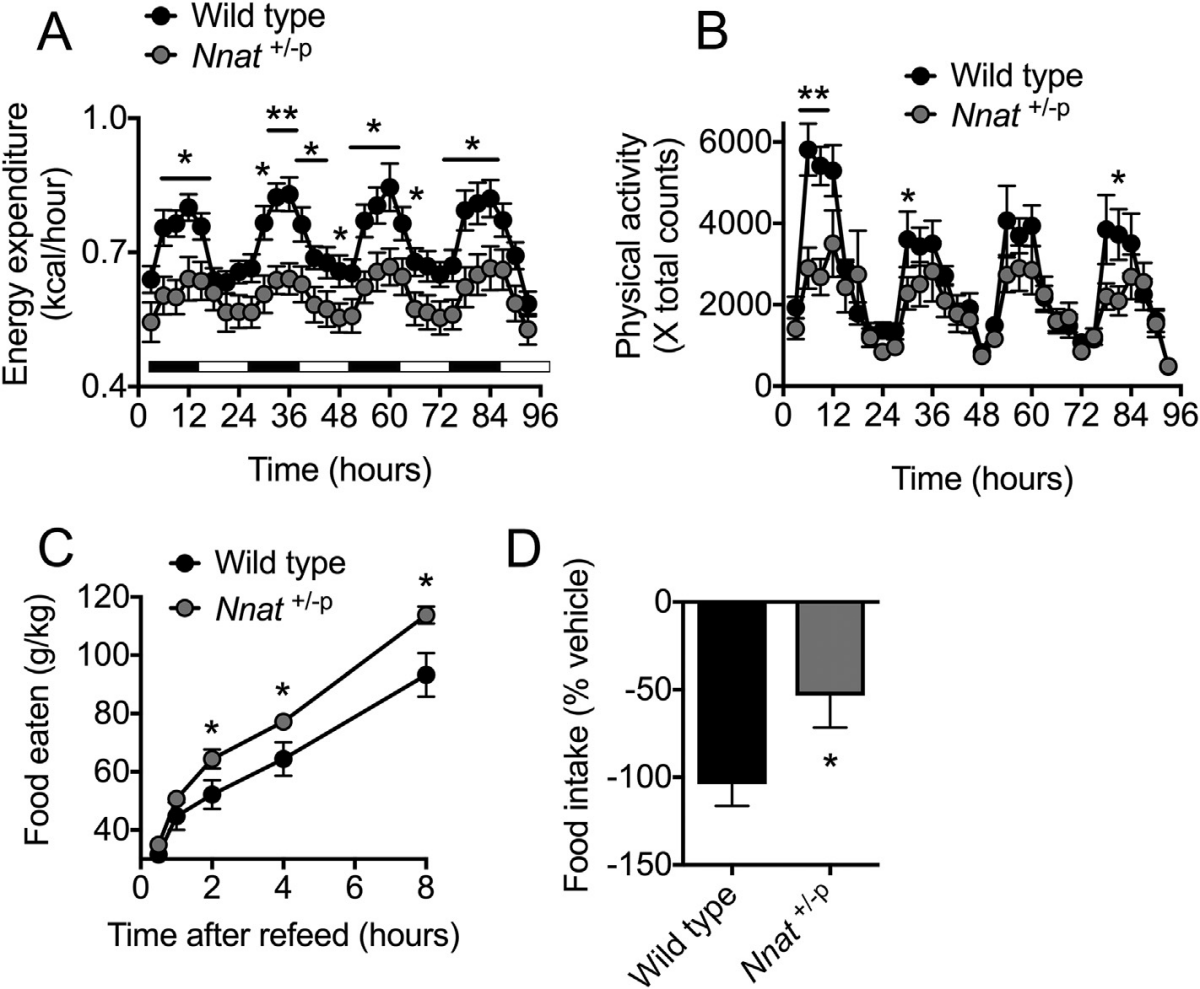
**Adult *Nnat* null mice on a 129S2/Sv background display hyperphagia and reduced energy expenditure.** (**A**) Energy expenditure calculated by indirect calorimetry using CLAMS over a period of 96 h in young adult (P70) wild type and *Nnat*^+/−p^ male mice (*n* = 8 mice per genotype). Black bars indicate periods of dark cycles. (**B**) Physical activity in the same mice as **A** expressed as horizontal beam breaks over time. (**C**) Food intake following an overnight fast in wild type and *Nnat*^+/−p^ male mice (*n* = 10 animals per genotype). (**D**) Food intake alterations (percentage change) in response to exogenous leptin compared to saline crossover control in wild type and *Nnat*^+/−p^ male mice (*n* = 12 animals per genotype). In all panels, data represented as mean ± SEM (*p < 0.05, **p < 0.01).

*Nnat* is expressed in key hypothalamic neurons governing energy homeostasis, where it is regulated by nutrient supply and also leptin [28], [39], [40], [41]. Adult male *Nnat*^+/−p^ mice (P70) on a 129S2/Sv background (at which point they had reached the same body weight as their wild type littermates (Supplemental Figure 1H)) had reduced energy expenditure as measured by indirect calorimetry (Figure 3A). This difference was most pronounced during the more active night cycle (Figure 3A). *Nnat*^+/−p^ mice also had lower levels of physical activity, as measured by horizontal beam breaks over the same time period, which was again more prominent during the night cycle, and also in the novel environment (Figure 3B). Despite no difference in *ad libitum* food intake over a 3-day period, feeding during the 8-hour period immediately following an overnight fast was increased in mutant mice (Figure 3C, Supplemental Figure 3A). Furthermore, whereas peripheral leptin administration in wild type mice resulted in substantially decreased food intake over a 3-day period, this effect was severely blunted in *Nnat*^+/−p^ mice (Figure 3D). This reduction in leptin sensitivity occurred despite unaltered levels of endogenous leptin in the serum of mutant mice at P70 (Supplemental Figure 3B). Together this indicates that adult *Nnat* deficient mice have lower energy expenditure, reduced sensitivity to the anorexigenic effects of leptin, and are hyperphagic.

### 3.4 Deletion of *Nnat* worsens age- and diet induced-obesity

**Figure 4:**
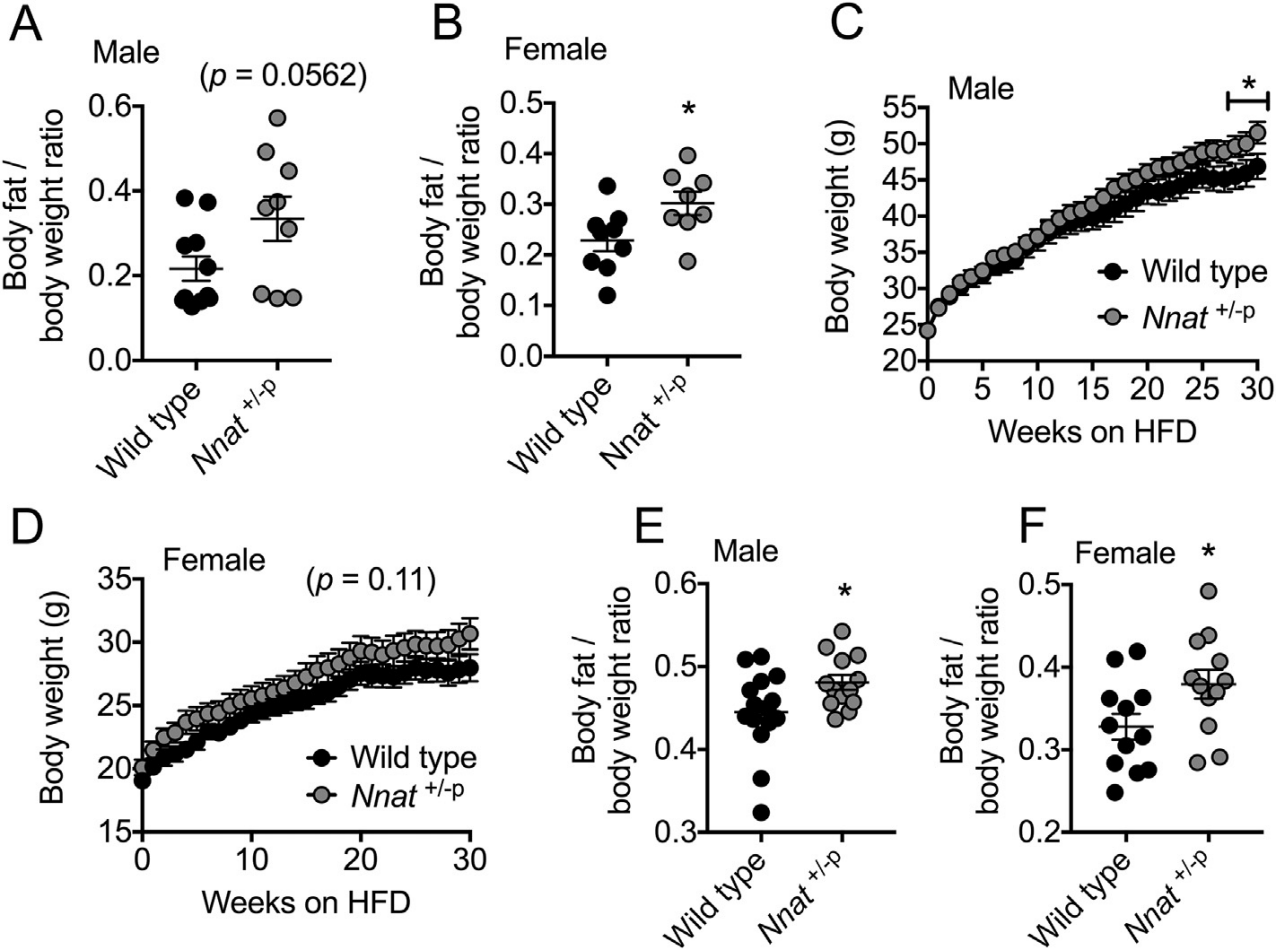
***Nnat* deficient mice on a 129S2/Sv background are more prone to obesity upon aging or HFD-feeding.** (**A**, **B**) Adiposity in male (A) and female (B) wild type and *Nnat*^+/−p^ mice was quantified by MRI scanning in chow-fed, 400 day old mice (P400) (*n* = 11 and 9 for wild type and *Nnat*^+/−p^ male mice respectively, *n* = 9 and 8 for females). (**C**, **D**) Body weights of male (C) and female (D) wild type and *Nnat*^+/−p^ mice fed high fat diet (HFD) for 30 consecutive weeks (*n* = 15 and 13 mice per genotype respectively for males, *n* = 12 mice for females). (**E**, **F**) Adiposity in male (E) and female (F) wild type and *Nnat*^+/−p^ mice was quantified by MRI scanning in mice fed high fat diet for 30 weeks (HFD) (*n* = 15 and 13 mice per genotype respectively for males, *n* = 12 mice for females). In all panels, data represented as mean ± SEM (*p < 0.05).

Restricted postnatal growth and development have been shown to influence health status in adult life with an increased risk of metabolic diseases such as obesity and diabetes [26], [27]. Additionally, single nucleotide polymorphisms in the human *NNAT* locus are associated with extreme childhood obesity, and reduced *NNAT* expression has been reported in the adipose tissue of obese children [28], [42]. Both an early growth restriction (Figure 1, Figure 2) and perturbed hypothalamic leptin sensing (Figure 3) suggest that adult *Nnat*^+/−p^ mice may be susceptible to obesity in later life. When *Nnat* deficient mice of both sexes were aged to over a year old (P400) on a standard chow diet, *Nnat*^+/−p^ mice demonstrated increased adiposity, which was statistically significant in aged females (Figure 4A,B). When challenged with a HFD, male *Nnat*^+/−p^ mice gained more weight than wild type littermates over the feeding period (Figure 4C). HFD-feeding of female *Nnat*^+/−p^ mice displayed the same pattern, although did not reach statistical significance (*p* = 0.11, Figure 4D). *Nnat*^+/−p^ mice of both sexes also accumulated more body fat as a ratio of body weight than HFD-fed wild type controls (Figure 4E,F).

### 3.5 *Nnat* deficient mice have reduced pituitary GH content and secretion

**Figure 5:**
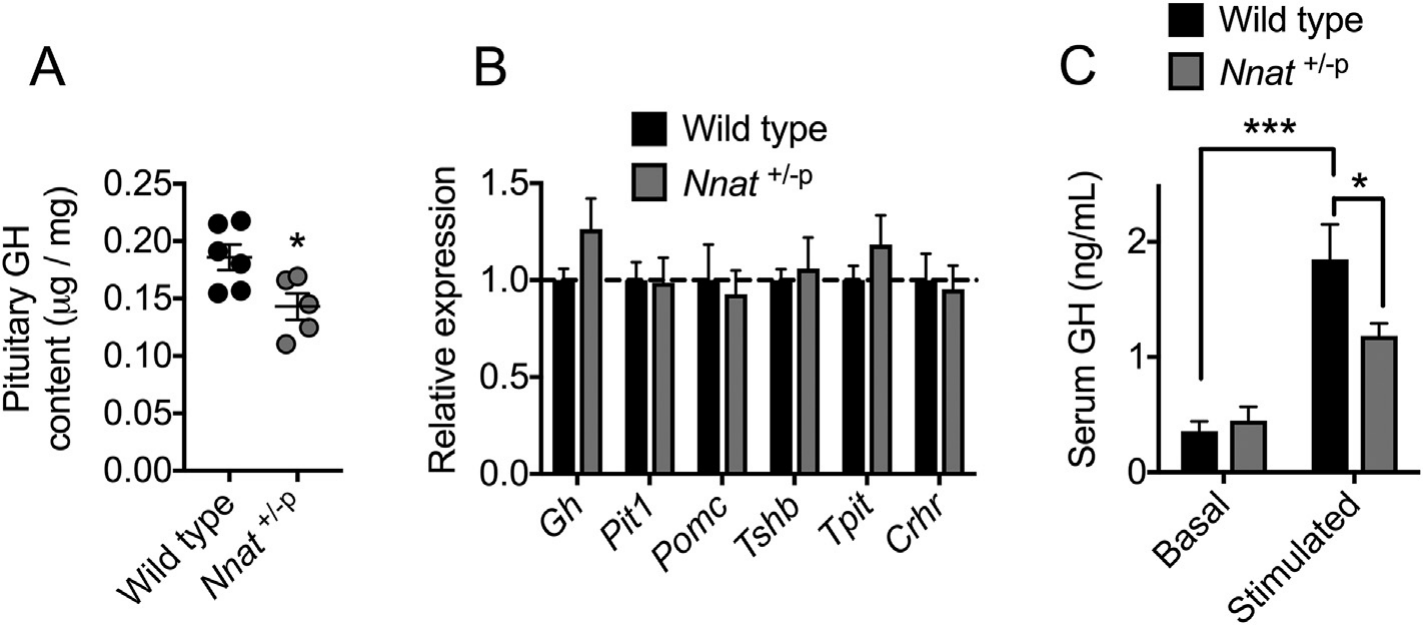
***Nnat* deficient mice on a 129S2/Sv background have defective pituitary gland growth hormone content and secretion.** (**A**) Growth hormone (GH) content was quantified in whole pituitary gland from wild type and *Nnat*^+/−p^ mice and normalized to total protein (*n* = 6 for wild type, *n* = 5 for *Nnat*^+/−p^). (**B**) Quantitative RT-PCR analysis of mRNAs encoding key genes in the pituitary gland of wild type and *Nnat*^+/−p^ mice. *Hprt* mRNA expression was used as an internal control and data is represented relative to wild type mice (*n* = 6 animals per genotype). (**C**) Measurement of GH secretion *in vivo* under basal and stimulated (hypoglycemic) conditions in wild type and *Nnat*^+/−p^ male mice (*n* = 14 animals per genotype). In all panels, data expressed as mean ± SEM (*p < 0.05, ***p < 0.001).

Our recent findings have indicated that NNAT interacts with the SPC in pancreatic beta cells, and that this is critical for maintaining adequate storage and secretion of mature insulin [41]. Circulating insulin is required for accumulation of adipose tissue [56], [57], and defects in the postprandial elevation of insulin may contribute to the growth defects and reduced adipocyte mass seen early in the early postnatal period in *Nnat*^+/−p^ mice. We also reasoned that alterations in the processing of other hormones may contribute to these abnormalities. Therefore, we assessed the content and secretion in *Nnat*^+/−p^ mice of GH, as this is another key circulating hormone crucial for growth and adipose accumulation in the postnatal period [58], [59] and neuronatin is expressed at high levels in the anterior pituitary [34]. GH content was appreciably reduced in the pituitary of null mutant mice compared to wild type littermates (Figure 5A). This was in the absence of any reduction to expression of GH mRNA, or changes in the mRNA expression of other key markers in the pituitary including *Pit1*, *Pomc*, *Tshb*, *Tpit*, and *Crhr* (Figure 5B). Alongside this reduced GH content, and similar to our findings for blunted glucose-evoked insulin secretion in *Nnat*^+/−p^ mice, pituitary GH secretion into the blood under hypoglycemia-stimulated conditions was severely reduced in these null animals (Figure 5C).

## 4 Discussion

Imprinted genes regulate growth and development in early life, both *in utero* and in the postnatal period. Altered imprinted gene expression in both human disease states and in mouse genetic models demonstrates the importance of their dosage and function, with an array of metabolic disorders stemming from inappropriate expression levels of imprinted genes [17], [18], [19], [20], [22], [23], [24], [25]. Moreover, abnormal growth in the postnatal period, due to genetic or environmental causes (such as reduced or excessive nutrient availability) is itself strongly associated with the risk of developing metabolic disease in later-life [26], [27].

Previous data has suggested that the imprinted gene *Nnat*, is associated with human obesity [28], [42]. It has also been suggested that *Nnat* forms part of a TRIM28-dependent imprinted gene expression network and that this is involved in the stochastic obesity seen in in-bred mouse strains. *Nnat* null mice have thus been reported to display a bimodal body weight phenotype with normal and obese subpopulations [42]. We also detected, in our own targeted allele of neuronatin, a bimodal body weight phenotype on a C57BL/6J background, albeit with a lean rather than obese subpopulation of mutant mice. Therefore, this genetic manipulation did not result in any significant obesity phenotype on this background, and we found no evidence for any underlying changes to energy homeostasis such as feeding and leptin sensitivity. Moreover, when examined on a 129S2/Sv background, we found little evidence for bimodal body weight distribution in *Nnat* null mice. Therefore, while both studies hint that loss of neuronatin is associated with some phenotypic variation with respect to body weight on a C57BL/6J background this does not seem to be a robust observation. The reasons underlying the discrepancies between our findings and those of Dalgaard et al. [42] remain to be elucidated. They could reflect subtle variations in the study environment (such as housing conditions, diet and microbiota effects) or differences in the targeting approaches and genetic background in the two distinct *Nnat* null mouse models used.

The surprising lack of body weight phenotype at either postnatal or adult stages on a C57BL/6J background prompted us to study these parameters in mice on the 129S2/Sv background. This strain has previously revealed early growth defects in mutant mice that were not apparent on the C57BL/6J background [44], [45], [46], [47], [48]. Deletion of *Nnat* on the 129S2/Sv background resulted in growth restriction in the early postnatal period with subsequent catch up growth after weaning, a defect that was absent on C57BL/6J background [41]. Following this catch up period, adult 129S2/Sv *Nnat* null mice had decreased energy expenditure, blunted leptin sensitivity and hyperphagia, and were more susceptible to both aging and HFD-induced obesity in later-life. The present studies demonstrate the importance of the imprinted gene neuronatin in early postnatal growth, particularly on different genetic backgrounds. Furthermore, loss of neuronatin subsequently affects feeding behavior and energy expenditure, and therefore susceptibility to obesity, in adulthood.

Imprinted genes are expressed in key tissues governing resource allocation such as the placenta, WAT, and hypothalamus. Growth rates both before birth and in the immediate postnatal period can have a strong causal effect on metabolic phenotypes, with intrauterine and postnatal growth restriction increasing the likelihood of developing obesity in later life [26], [60]. This occurs when a developing organism responds to substandard environmental conditions during early stage programming. Ultimately, this leads to an increased risk of developing the metabolic syndrome in later life, a phenomenon known as metabolic programming [61], [62]. Proposed mechanisms for how this early-life adversity can be detrimental to long-term metabolic health include permanent changes in organ structure and programmed epigenetic changes in gene expression [63], [64]. Indeed, there is evidence that individual imprinted (*Peg3*, *Pref1/Dlk1*, *Gnasxl*, *Cdkn1c*, and *Grb10)* and also non-imprinted (*Gck*) genes can regulate both prenatal growth and postnatal energy regulation [8], [65], [66], [67], [68]. We did not detect *Nnat* expression in the placenta at various embryonic stages, in agreement with previous findings [69]. We also found no evidence for fetal growth restriction or placental defects in *Nnat* null embryos indicating that, at birth, these mutant mice are phenotypically indistinguishable from their wild type littermates suggesting that neuronatin does not play a major role in fetal growth.

Generally, paternally expressed genes enhance fetal and/or postnatal growth. Although fetal growth was normal in *Nnat* null embryos, we found that these animals developed a postnatal restriction in growth while still in the nest, which worsened from P10 until weaning age (P20). Postnatal mammals must adapt to independent life during this period, maintaining their core body temperature, beginning to acquire food other than that provided by their mother, and regulating their own metabolism. Although core body temperature was unaltered in *Nnat* null mice, growth restricted mutant mice had disproportionate reductions in adipose tissues when measured as percentage of body weight, in contrast to proportionate reductions in weights for other major organs. We show that NNAT is expressed in mature white adipocytes, and indeed previous *in vitro* studies have demonstrated that *Nnat* is able to potentiate adipogenesis [29]. After weaning, this body weight difference diminishes between *Nnat* deficient juveniles and their wild type littermates. This so-called ‘catch up’ growth acts to restore the size of the organism back to their ‘normal’ growth trajectory (reviewed in [26]) and is a common observation upon genetic modification of imprinted gene dosage in young mutant mice, often most prominent between P7 and P21 (reviewed in [70]). These mouse models with altered expression of imprinted genes demonstrating a failure to thrive in the early postnatal period mimics the pattern observed in human imprinting disorders such as Prader–Willi and Silver–Russell syndrome [19], [20].

Modification of imprinted gene dosage for various paternally expressed genes is also a risk factor for obesity in later life. Adult mice deficient in *Necdin* (*Ndn*) or mice with overexpression of *Mest* (*Peg1*) are obese owing to increases in adipose expansion [71], [72]. Obesity also results from deletion of paternally expressed genes, *Peg3*, *Pref1/Dlk1*, or *Magel2* with observed differences in hyperphagia and energy expenditure, all of which demonstrate catch up growth in null mice [66], [67], [73]. In our young adult *Nnat* null mice, we find a combination of reduced energy expenditure, reduced physical activity, hyperphagia, and partial leptin resistance, at which point null mice had reached the same body weight as their wild type littermates. These metabolic alterations were consistent with subsequently worsened obesity in aged, or HFD-fed, *Nnat* mutant mice. The fact that body weight and adiposity are unaltered in adult (P70) *Nnat* null mice suggests that the defective leptin sensitivity observed in these mice is a primary defect, rather than being secondary to the development of obesity. Indeed, *Nnat* is expressed in leptin receptor-expressing neurons in the hypothalamus that dictate feeding behaviour and energy expenditure and is also upregulated upon leptin administration in mice [28], [74].

We have recently shown that *Nnat* is able to potentiate signal peptidase processing of preproinsulin and that loss of *Nnat* expression in pancreatic beta cells results in defective insulin storage and therefore glucose-evoked insulin secretion, which is particularly prominent under conditions of nutrient excess [41]. It is therefore possible that the intensive period of growth and development during the postnatal phase (when expression of *Nnat* peaks) requires *Nnat*-mediated increases in insulin secretion to maintain this growth demand, and adequate adipose accumulation. Clearly, circulating insulin is required for accumulation of adipose [56], [57] and indeed, postnatal exogenous insulin administration in rodents results in greater body weight gain [75], [76]. Furthermore, evidence that insulin action plays a much greater role in postnatal rather than prenatal growth is demonstrated by the fact that mice with either *Ins1* or *Ins2* heterozygosity are born with only a slight growth defect and are metabolically healthy. This is in contrast to the deterioration of glucose homeostasis that occurs in the postnatal period in these animals [77]. Insulin secretion in our postnatal or adult *Nnat* null mice was not reduced to the point where we detected any changes in glucose homeostasis although this may be background-dependent, as mice with insulin signalling defects on a 129S2/Sv background display minimal defects in glucose homeostasis [78]. However, it is clear that neuronatin is critical for precise matching of insulin production and secretion to nutrient availability to permit normal responses to increased energy intake. Therefore, loss of neuronatin may perturb appropriate deposition of lipids due to dysregulated post-prandial insulin secretion. In addition to our documented role in insulin secretion via effects on early peptide handling [41], we further demonstrate in this work that neuronatin is also required for adequate storage and secretion of pituitary GH *in vivo*. GH is another anabolic hormone with a key role in postnatal growth and adipose accumulation [58], [59]. It therefore appears that in the immediate postnatal phase, in which growth rates are particularly high, that *Nnat* is required to match the demand for increased production of circulating hormones such as insulin and GH.

Neuronatin is expressed in the hypothalamus and adipose tissue and our current studies do not rule out a role for neuronatin action directly in these key regulatory tissues in the growth and metabolic defects we observe. However, to fully elucidate the tissue-specific roles of neuronatin in adipose tissues and various hypothalamic nuclei will require extensive further work with mice with loss of function in specific cell populations.

In summary, this work demonstrates that neuronatin deletion in mice on a 129S2/Sv background causes a transient postnatal growth restriction with subsequent catch up growth following weaning. In adulthood these animals display numerous obesogenic phenotypes that culminate in increased susceptibility to obesity from either aging or high fat feeding. Collectively, these data demonstrate the importance of the imprinted gene neuronatin in postnatal growth, adult energy homeostasis, and the pathogenesis of obesity.

## Author contributions

S.J.M., J.S., and D.J.W. designed the research; S.J.M., S.J.T., M.V.D.P., A.I.C., and E.E.I. performed experiments; S.J.M., S.J.T., R.M.J., and D.J.W. drafted and/or wrote the manuscript; M.C., A.G.F., J.S., and D.J.W. provided funding; J.S. and D.J.W. supervised the work.

## Figures and Tables

**fig s1:**
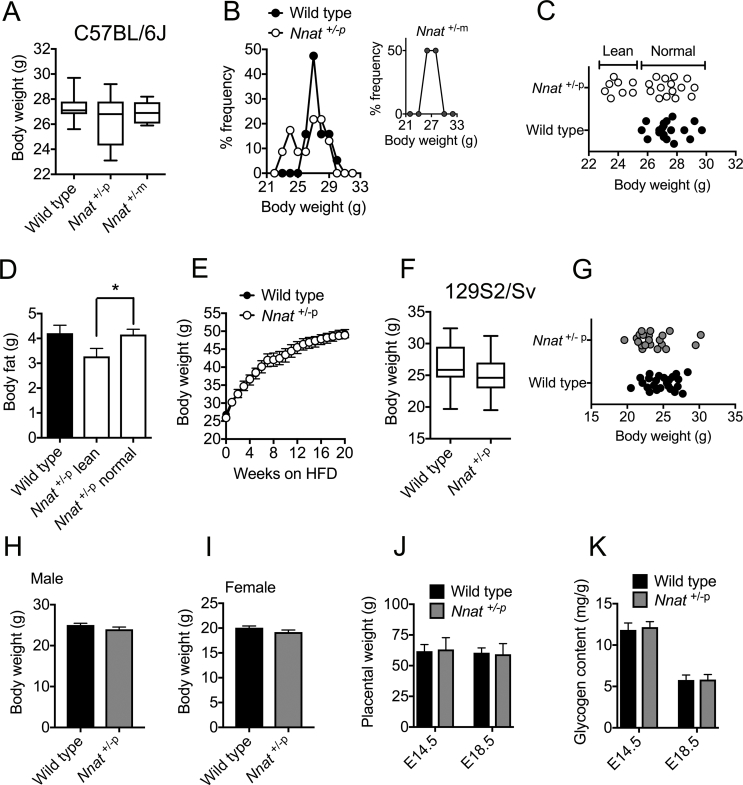
1

**fig s2:**
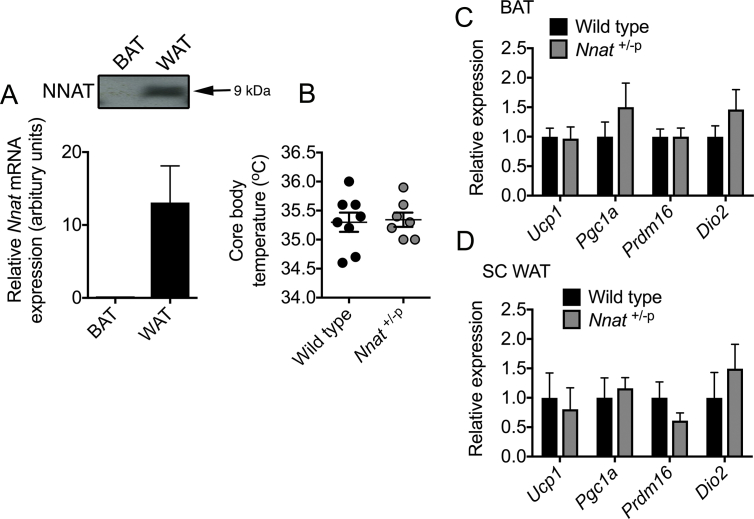
2

**fig s3:**
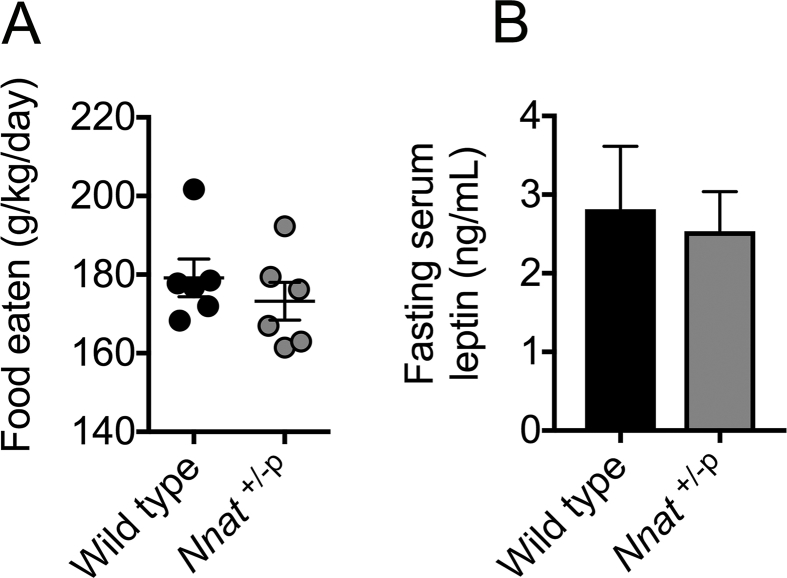
3
