# Supplementary material for: Neuronatin deletion causes postnatal growth restriction and adult obesity in 129S2/Sv mice
Source: Mol Metab. 2018 Sep 15;18:97–106. doi: 10.1016/j.molmet.2018.09.001 (PMC6308027; doi:10.1016/j.molmet.2018.09.001)
Supplement: Multimedia component 1 [file mmc1.docx]

**Supplemental figure legends**

**Supplemental Figure 1. Bimodal and placental analysis upon *Nnat* deficiency**. (**A-C**) Body weights of 12 week old wild type, *Nnat*^+/-p^ and *Nnat*^+/-m^ male mice on C57BL/6J background represented as hypervariability by box and whisker plot (A), frequency distribution (B) and as individual body weights showing lean and normal subpopulations (C). Inset in B shows frequency distribution in *Nnat*^+/-m^ mice (*n* = 19, 23 and 8 for wild type, *Nnat*^+/-p^ and *Nnat*^+/-m^ mice respectively). (**D**) Adiposity in wild type and both lean and normal subpopulations of *Nnat*^+/-p^ male mice on C57BL/6J background quantified by MRI scanning (*n* = 9 mice per group, * *p* < 0.05). (**E**) Body weights of wild type and *Nnat*^+/-p^ male mice on C57BL/6J background fed HFD for 20 weeks. (**F**, **G**) Body weights of 12 week old wild type and *Nnat*^+/-p^ male mice on 129S2/Sv background represented as hypervariability by box and whisker plot (F) and as individual body weights (G) and (*n* = 24 and 21 for wild type and *Nnat*^+/-p^ mice respectively). (**H, I**) Body weights of male (H) and female (I) wild type and *Nnat*^+/-p^ (paternal deletion) mice on 129S2/Sv background at 10 weeks old (P70) (*n* = 19 and 20 for wild type and *Nnat*^+/-p^ mice respectively, *n* = 17 and 18 for females). Data in panels A-I is represented as mean ± SEM. (**J**) Wet weights of placentas from wild type and *Nnat*^+/-p^ embryos on 129S2/Sv background at E14.5 and E18.5 timepoints (*n* = 20 and 16 for E14.5 and E18.5 respectively per genotype). Data is represented as mean ± SD. (**K**) Glycogen content of placentas in J normalized to total placental weight (*n* = 10 per genotype, per embroyonic time point). Data is represented as mean ± SD.

**Supplemental Figure 2. Thermogenic markers in adipose of *Nnat* deficient mice on a 129S2/Sv background.** (**A**) Quantitative RT-PCR analysis of *Nnat* mRNA in BAT and WAT of wild type mice. *Cyclophilin A* mRNA expression was used as an internal control (*n* = 4 mice). Inset shows Western blot analysis of the same tissues using antibodies against NNAT. (**B**) Core body temperatures of wild type and *Nnat*^+/-p^ male mice at weaning age (P20) (*n* = 8 and 7 for wild type and *Nnat*^+/-p^ mice respectively). (**C**, **D**) Quantitative RT-PCR analysis of thermogenic markers in BAT and subcutaneous (SC) WAT of P20 *Nnat*^+/-p^ mice relative to expression in tissues of wild type mice (*n* = 5 mice per genotype). *Cyclophilin A* mRNA expression was used as an internal control. Data in all panels is represented as mean ± SEM.

**Supplemental Figure 3. *Ad libitum* food intake and serum leptin levels in *Nnat* deficient adult mice on a 129S2/Sv background.** (**A**) *Ad libitum* feeding in adult (P70) wild type and *Nnat*^+/-p^ male mice as measured over a 3-day period (*n* = 6 animals per genotype). (**B**) Fasting serum leptin levels in wild type and *Nnat*^+/-p^ mice at P70 (*n* = 8 and 10 animals respectively). Data in both panels is represented as mean ± SEM.

| **Supplemental Table 1.** | |
| --- | --- |
| **Probes for RT-PCR (TaqMan, Applied Biosystems, all *Mus Musculus*)** | |
| *Nnat* | Mm00440480_m1 |
| *Hprt* | Mm00446968_m1 |
| *Cyclophilin A* | Mm03302254_g1 |
| *Ucp1* | Mm01244861_m1 |
| *Pgc1a* | Mm00447183_m1 |
| *Prdm16* | Mm00712556_m1 |
| *Dio2* | Mm00515664_m1 |
| *Gh* | Mm01258409_g1 |
| *Pit1* | Mm00476852_m1 |
| *Pomc* | Mm00435874_m1 |
| *Tshb* | Mm00437190_m1 |
| *Tpit* | Mm00453377_m1 |
| *Crhr* | Mm00432670_m1 |

| **Supplemental Table 2. Sequences of primers used for**  **PCR amplification of PEG expression in placenta** | | |
| --- | --- | --- |
| **Target** | **Forward** | **Reverse** |
| ***Nnat*** | 5’-GAACTGCTCATCATCGGCTG-3’ | 5’-AAGGGGAAATGAGGGAGCAA-3’ |
| ***Peg3*** | 5’- CCAAGAGAACTGCCTACCCA-3’ | 5’-TGGTCTCCTCATCCTCCTGA-3’ |
| ***Peg6*** | 5’- CAACCGTATGCCCATGACAG-3’ | 5’-GTCCTGCTAACTCTCCAGGG-3’ |
| ***Peg10*** | 5’-TGGATCAGGTGGAGTTGCTT-3’ | 5’-AAGGCTCTGGGTGAAGGATC-3’ |
